# The ability of seeds to float with water currents contributes to the invasion success of *Impatiens balfourii* and *I. glandulifera*

**DOI:** 10.1007/s10265-020-01212-0

**Published:** 2020-07-03

**Authors:** Kamil Najberek, Paweł Olejniczak, Katarzyna Berent, Magdalena Gąsienica-Staszeczek, Wojciech Solarz

**Affiliations:** 1 Institute of Nature Conservation, Polish Academy of Sciences, Al. Adama Mickiewicza 33, 31-120 Kraków, Poland; 2 Academic Centre for Materials and Nanotechnology, AGH University of Science and Technology, Al. Adama Mickiewicza 30, 30-059 Kraków, Poland; 3 Zakopane, Poland

**Keywords:** Biological invasions, Invasion corridors, Lag phase, Seed coat thickness, Seed cross-section, Tetrazolium (TZ) test

## Abstract

Two alien species in Europe, *Impatiens glandulifera* and *I. balfourii*, are closely related, have similar growth rates and reproductive capacities, and are very attractive to pollinators. Nevertheless, only *I. glandulifera* is a highly invasive alien species in Europe, while *I. balfourii* is non-invasive. We assumed that the varying levels of invasiveness are driven by differences in the floating ability of their seeds, which may determine the invasion success of riparian alien plants, such as the *Impatiens* species. By mimicking two types of aquatic conditions, we determined seed floating ability for each species from younger and older populations. We also analyzed four seed traits: seed viability, surface, shape and coat structure. Seeds of the non-invasive *I. balfourii* float less well than seeds of the invasive *I. glandulifera*. We also found that the seeds of *I. balfourii* from the younger population have a higher floating ability in comparison with that of the seeds from the older population. The results for *I. glandulifera* were the opposite, with decreased floating ability in the younger population. These differences were associated with seed surface, shape and coat structure. These results indicate that the floating ability of *I. balfourii* seeds may increase over time following its introduction into a given area, while in the case of *I. glandulifera*, this ability may gradually decrease. Therefore, the former species, currently regarded as a poor disperser, has the potential to become invasive in the future, whereas the latter does not seem to benefit from further investments in the floating ability of its seeds.

**Electronic supplementary material:**

The online version of this article (10.1007/s10265-020-01212-0) contains supplementary material, which is available to authorized users.

## Introduction

Identifying the factors that determine the invasive behaviors of species introduced to new areas is an increasingly important task both for theoretical aspects of ecology and for practical implications in nature conservation and economics (Vazquez and Morales 2011). Several noteworthy invasions in different parts of the world make the *Impatiens* genus extremely harmful (Janssens et al. 2009; Vervoort et al. 2011). Despite the large number of studies addressing alien *Impatiens* species (e.g., Čuda et al. 2014; Elst et al. 2016; Jacquemart et al. 2015; Janssens et al. 2009; Kollmann and Bañuelos 2004; Tanner et al. 2015; Ugoletti et al. 2011; Vervoort et al. 2011), there are still knowledge gaps regarding the great differences in the levels of invasiveness exhibited by closely related representatives of this genus introduced to the same region. Examples of such species are *Impatiens glandulifera* and *I. balfourii* (Janssens et al. 2009), where the former is a highly invasive alien species in Europe and the latter is non-invasive (Najberek et al. 2018).

Previous comparative studies of *I. balfourii* and *I. glandulifera* demonstrated that they are similar in terms of their photosynthetic capacities, growth rates (Ugoletti et al. 2011), attractiveness to pollinators, self-compatibility, high reproductive capacity (Jacquemart et al. 2015; Ugoletti et al. 2011) and lack of inbreeding depression (Jacquemart et al. 2015). Thus, the invasiveness of these two species should be similar, but it is not. The low invasiveness of *I. balfourii* has been explained mainly by its low frost tolerance (Perrins et al. 1993; Tabak and von Wettberg 2008). However, it is also thought that invasion by this species may still be in a lag phase, as it was introduced 60 years later than its invasive counterpart (Adamowski 2009). Nevertheless, neither of these two hypotheses has ever been verified.

In the present study, it has been assumed that the varying levels of invasiveness of the two *Impatiens* species may be partly driven by differences in the ability of their seeds to float with water current. This ability is thought to favor the success of annual plants that grow along rivers, such as *Impatiens* species, after their introduction into new areas (Pyšek and Prach 1993). An increase in the dispersal of species may also occur along human-made structures that allow water flow, such as road drainage systems. However, differences in floating abilities have not been verified using alien species varying in invasiveness.

In our earlier study (Najberek et al. 2017), we demonstrated that *I. balfourii* occurrence along roadsides in Swiss-Italian Insubria may be connected to the lower pressure of natural enemies. At the same time, this habitat may in fact be a trap for *I. balfourii* because of the very frequent mowing of roadsides on the Swiss side in comparison to the Italian side. This phenomenon was also demonstrated on the European scale, in which six populations of *I. balfourii* from Croatia, Italy, France and Andorra were surveyed (Najberek et al. 2020). On the other hand, the other species included in the study, *I. glandulifera*, prefers moist areas, such as riversides and/or wetlands (Helmisaari 2010). In terms of human impact, moist habitats are less frequently disturbed and therefore may be “safer” for plants than roadsides. Additional advantages include the high propagule pressure, reduced competition with native species, and wide range of available microhabitats along water courses (Čuda et al. 2017; Hufbauer et al. 2012; Planty-Tabacchi et al. 1996; Stromberg et al. 2007). The present study is the first to investigate the floating ability of seeds in order to identify the drivers of potential differences in habitat preferences between the two *Impatiens* species. Currently, it is known that only *I. glandulifera* seeds can float in still waters for as long as forty days (Tabak and von Wettberg 2008), whereas the floating ability of *I. balfourii* seeds has not yet been tested.

The post-introduction changes during biological invasion received little attention from scientists until the 2000s. Today, it seems obvious that the process enables alien species to expand into regions and become established in habitats that were previously inaccessible (Barrett et al. 2016; Cox 2004; Gruntman et al. 2017; Monty and Mahy 2010; Niklas 2016; Phillips et al. 2006; Sax et al. 2005; Walther et al. 2009). These changes are a result of each alien species adapting in response to interactions with specific local communities, which are formed from both native species and alien species that had been introduced earlier, as well as in response to the local abiotic environment (Gioria and Osborne 2014; Mooney and Cleland 2001). Other characteristics of introduction events, such as the number of introduced individuals or their sex ratio, may also be major determinants of the future dynamics of the traits. In this study, we compared seeds from younger and older populations of *I. balfourii* and *I. glandulifera*. We hypothesized that seeds from younger populations of each species float better than seeds from older populations, where their superior better floating ability provides them with higher dispersal potential, hence influencing their invasion success. Because *I. glandulifera* prefers moist areas and its range is expanding, the above assumption should be more applicable to this species. On the other hand, *I. balfourii* prefers roadsides to riversides, and therefore, if the seeds from its younger populations float better than those from its older populations, then the dispersal potential of this non-invasive alien species may notably increase in the near future.

We tested the hypotheses in still and turbid water conditions, analyzing factors potentially influencing the ability of seeds to float. The floating ability of seeds may change with their size and shape; therefore, the surface area, weight, circularity and aspect ratio of seeds were assessed. The analyzed seed structure features accounting for floating ability included the presence of air-filled “cavities” in the seed coat, the thickness of the outer layer of the seed coat, and the levels of calcium and oxygen-containing compounds.

## Materials and methods

### Species selected for study

Both species were introduced to Europe from the western Himalayas as ornamentals: *I. balfourii* in 1901 (Fournier 1952) and *I. glandulifera* in 1839 (Valentine 1971). Their habitat preferences differ; nonetheless, they are highly adaptable and may occur in the same areas (Ugoletti et al. 2013). *Impatiens balfourii* prefers a drier substrate, while *I. glandulifera* occurs more frequently in moist habitats (Najberek et al. 2018). Both species have similar flower morphology and disperse their seeds ballistically (Jacquemart et al. 2015). In Europe, they prefer ruderal areas highly altered by humans (e.g., roadsides, train tracks) and seminatural and natural sites, such as riversides and forest edges.

### Study area and seed collection

The study focused on testing the floating ability of seeds of the two *Impatiens* species from populations of different ages; therefore, it was critical to choose two related populations for each species, including older parental and younger descendant populations (Table 1), which were chosen based on data in the literature. Earlier studies conducted on *I. glandulifera* (Gruntman et al. 2017) demonstrated that the effects of post-invasion changes can be detected as early as 20 years after the initial introduction. Therefore, there was at least a 20-year age difference between the selected populations of the two *Impatiens* species.

**Table 1:** Characteristics of localities hosting the non-invasive alien species (‘NIAS’) *I. balfourii* and the invasive alien species (‘IAS’) *I. glandulifera* in Swiss-Italian Insubria, the Cracow area in Poland, and Istria in Croatia

Species	Region	Year of introduction (population age; references)	Habitat	Elevation [a.s.l.]	Coordinates	N seeds/experiment
*I. balfourii* (NIAS)	Insubria	1940s (older population; Info Flora 2019)	Roadside	322	8.766436 E, 46.00067 N	45/still water
			Roadside	207	8.694318 E, 46.0768 N	74/still water
			Riverside	256	8.753205 E, 46.04423 N	40/still water; 20/turbid water
			Roadside	216	8.731817 E, 46.06609 N	40/still water; 20/turbid water
			Roadside	268	8.792629 E, 45.99434 N	51/still water; 20/turbid water
			Roadside	273	8.756866 E, 46.18733 N	20/turbid water
			Roadside	219	8.74125 E, 46.10332 N	20/turbid water
	Istria	1970s (younger population; Slavko Brana, pers. comm. 2018)	Ruderal area	681	14.05362 E, 45.48411 N	110/still water; 40/turbid water
			Roadside	674	14.05328 E, 45.48425 N	30/still water; 10/turbid water
			Roadside	670	13.99924 E, 45.4982 N	60/still water; 20/turbid water
			Garden	672	13.99922 E, 45.49771N	50/still water; 30/turbid water
*I. glandulifera* (IAS)	Insubria	1960s (older population; Info Flora 2019)	Roadside	210	8.7463 E, 45.97466 N	51/still water; 25/turbid water
			Path edge	207	8.882878 E, 46.15888 N	99/still water; 25/turbid water
			Riverside	191	8.866396 E, 46.15549 N	100/still water; 27/turbid water
			Near recreation path	198	8.865667 E, 46.15174 N	23/turbid water
	Cracow	1980s (younger population; Zając et al. 2011)	Wetland/roadside	212	19.75073 E, 49.97861 N	80/still water; 30/turbid water
			Meadow at forest edge	379	19.62610 E, 49.92266 N	60/still water; 30/turbid water
			Roadside	244	19.87518 E, 50.08796 N	40/still water; 10/turbid water
			Riverside	207	19.84832 E, 50.07790 N	70/still water; 30/turbid water

The number of seeds used in particular experiments is shown

**Fig. 1:**
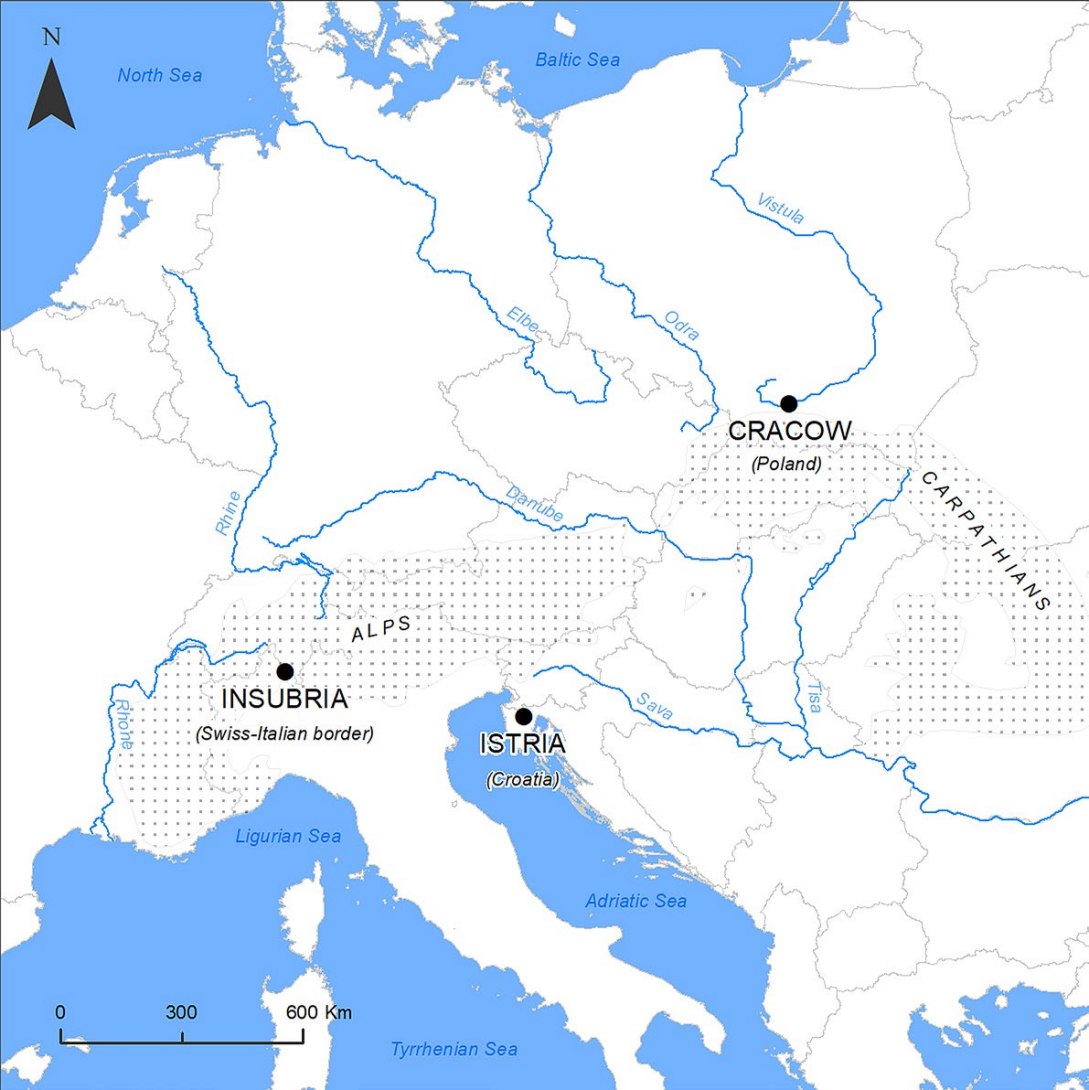
Surveyed populations of the non-invasive alien *Impatiens balfourii* (Insubria, Istria) and the invasive alien *I. glandulifera* (Insubria, Cracow). Insubrian populations of the two species are older, while the populations from Istria and Cracow are younger (see Table 1)

The following regions were studied: Swiss-Italian Insubria, the Cracow area in Poland, and Croatian Istria (Table 1, Fig. 1). *I. glandulifera* extended its range from Insubria in Switzerland through Germany to SW Poland (Helmisaari 2010; Pyšek and Prach 1993), from where it extended its range further to the east, including Cracow and surrounding areas (Tokarska-Guzik 2005; Zając et al. 2011). The expansion routes of *I. balfourii* are less clear, and it is not certain if this species extended its range from Insubria to the Croatian Istria through Italy and Slovenia or through Austria and Slovenia (Adamowski 2009). However, taking into account that the species prefers warmer areas, the first scenario seems to be more likely. Moreover, we found that the species was first recorded in Istria and subsequently extended its range into the continental part of Croatia (Slavko Brana, personal communication).

In each region, localities hosting *I. balfourii* and *I. glandulifera* were detected during the flowering period in September/October, when the plants are conspicuous and easy to find. The seeds of both species were collected in October 2016 in Insubria, in September 2017 in Cracow and its surrounding areas and in October 2017 in Istria.

The characteristics of the surveyed localities and the number of seeds used in particular experiments are presented in Table 1. The seeds were collected from various habitat types with the exception of *I. balfourii* from Insubria, where the seeds originated from mainly roadsides. However, in this region more than 80% of the localities of the species occurred along this habitat (Najberek et al. 2017), therefore, the proportion of selected localities (six roadsides and one riverside) reflects the actual frequency of both habitat types in this region. It should also be stressed that roads in Insubria are often bordered by high scarps; during rain events, the water flows down from the scarps and runs along the roads and/or in ditches. Thus, the seeds can be dispersed not only in natural waters of streams and lakes, but also along man-made road drainage systems.

### The ability of the seeds to float in still and turbid water

The ability of the seeds collected in Insubria to float in still water was assessed over a period of 62 days in 2016, while the seeds from Cracow and Istria were assessed over a period of 64 days in 2017. In both study years, the turbid water tests were carried out simultaneously with the still water tests. Nevertheless, the turbid water tests were less time consuming, taking only 1 day per study year.

The still water experiments were carried out with Erlenmeyer flasks (250 ml capacity; 20 flasks per species and 10 flasks per population); the flasks were filled with 200 ml of deionized water. Seeds of the same population from different localities (Table 1) were pooled and inserted randomly into the flasks (25 seeds per flask). The sunken seeds were counted once a day during the first 5 days of the experiment, two times during the second week and once a week until the end of the experiment.

Parallel tests of the ability of the seeds to float in turbid water were carried out using Erlenmeyer flasks (100 ml capacity; 20 flasks per species and 10 flasks per population) filled with 75 ml of deionized water and mounted in an orbital shaker (GFL 3015). Seeds of the same population from different localities (Table 1) were pooled within populations and inserted into the flasks (10 seeds per flask). After pre-defined periods of shaking, the shaker and the timer were paused, the sunken seeds were counted, and the shaking was resumed. The seeds were counted after every 20 s of shaking during the first 4 min, after every 60 s of shaking during the next 8 min, after every 120 s of shaking during the next 16 min, after every 240 s of shaking during the next 20 min, after every 480 s of shaking during the next 32 min and after every 960 s of shaking during the next 2 h and 8 min. The overall duration of the experiment was 3 h and 28 min. The same shaker set to the same parameters (frequency = 4 Hz, amplitude = 3 cm) was used in 2016 and 2017.

The water parameters were comparable across all experiments: temperature of 20 °C, conductivity of 8 µS cm^–1^, pH 8.5, 3.9 mg L^−1^ TDS, salinity of 0.01 psu, resistance of 1.14e + 5 Ω cm, redox potential of − 88 mV and 3.1e − 2 mol L^–1^ ions.

### Tetrazolium (TZ) test

**Fig. 2:**
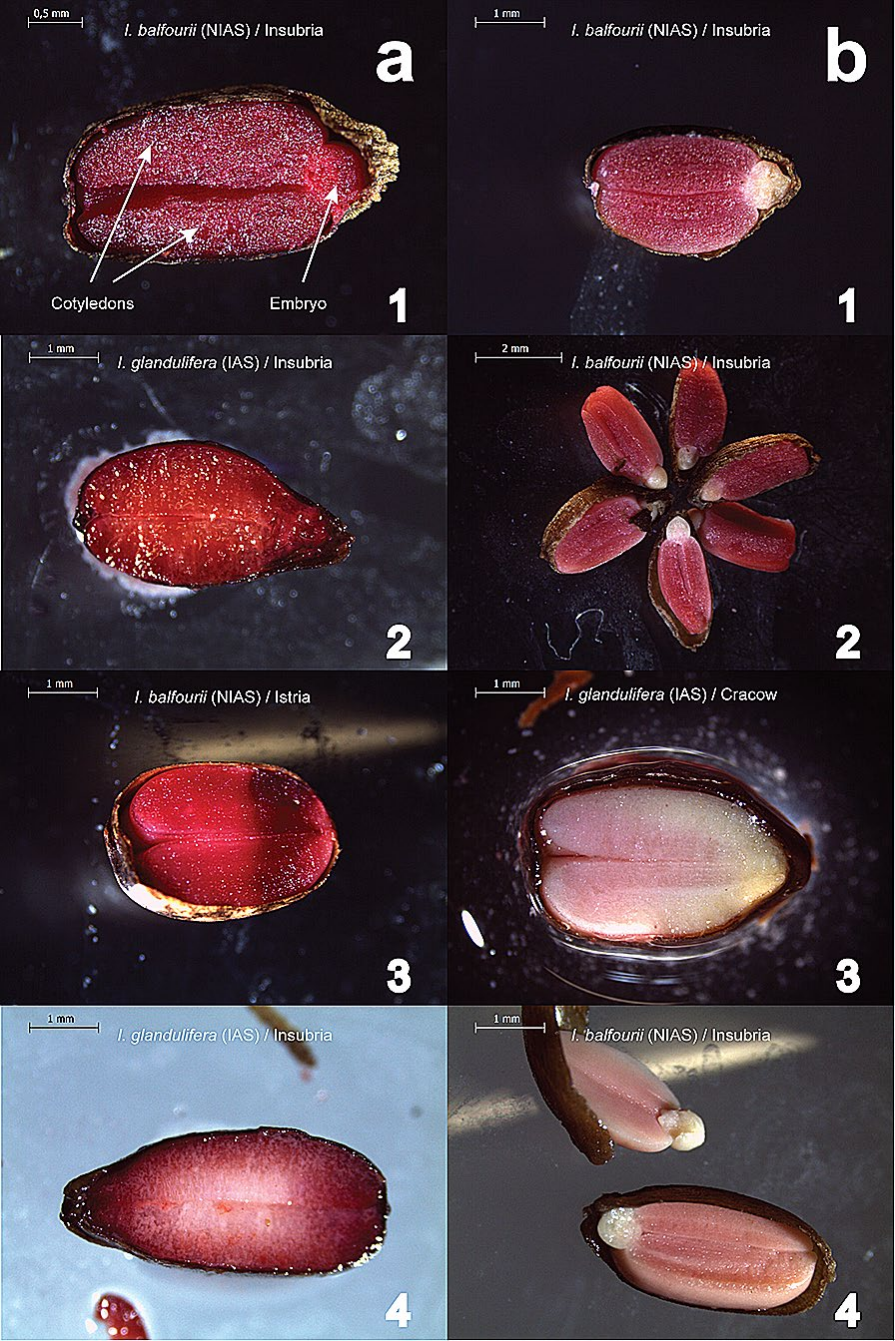
Examples of viable (**a**1–4) and unviable (**b**1–4) seeds. Seeds of the non-invasive alien species (‘NIAS’) *Impatiens balfourii* (**a**1, 3, **b**1, 2, 4) and the invasive alien species (‘IAS’) *I. glandulifera* (**a**2, 4, **b**3) from different populations were evaluated

Following the methods adapted for *I. glandulifera* (Van Meerbeek et al. 2015), the viability of seeds of both species was assessed with the TZ test. Seeds (*N* = 500) were hydrated for 24 h in deionized water at 20 °C on a Petri dish. After this period, the water was removed, and the seeds were rinsed once with deionized water. Subsequently, a 1% tetrazolium solution (pH ± 7) was prepared and used to stain the seeds for 24 h at 35 °C in tubes. Then, the tubes were closed and stored in a refrigerator. Before each assessment, the tubes were removed from the refrigerator. The seeds were rinsed three times with deionized water and cut into two halves. Their viability was evaluated under a stereomicroscope (model Leica S8 APO). The occurrence of a viable embryo (stained red) was an indicator that the seed was viable (Fig. 2a1–4), whereas seeds with a dead embryo (stained white) were classified as unviable (Fig. 2b1–4). Moreover, seeds with dead embryos were classified as unviable even if the cotyledons were viable (Fig. 2b1–2).

### Seed surface index (Ss), shape descriptors and structure assessment

Seeds were photographed (Canon EOS 60D, Canon EF 100 mm f/2.8 Macro USM lens and ring flashlight) and the digital images were analyzed with ImageJ software (ver. 1.51 k; Schneider et al. 2012). The software analyzed the area of symmetrical cross section of each seed, which corresponded to the total submerged surface. The seeds from particular localities (*N* = 6215) were weighed in 24 packages using a PS 360.R2 electronic scale with a weighing accuracy of 0.001 g. The Ss for particular localities was calculated as the ratio of the average seed area to the average seed weight (mm^2^ g^–1^).

Two types of shape descriptors were taken into account: the circularity and aspect ratio. The circularity, the parameter that shows how closely the shape approaches a circle, was calculated according to the formula 4π × seed area/seed perimeter^2^. The aspect ratio is the ratio of the major axis of the seed to its shorter minor axis. Both parameters allowed us to assess whether the studied seeds were oblong or rounded. As in the Ss analysis, the data on shape descriptors were obtained from the digital images analyzed with ImageJ software.

Surface structure was assessed through the analysis of 16 seeds (four seeds per studied population) by FEI Versa 3D field emission scanning electron microscope (FE-SEM). The structural differences of the plants were visualized using the secondary electron (SE) detector in the low vacuum (LV) mode at an acceleration voltage of 10 kV. LV-SEM enables the investigation of hydrated and nonconductive plant samples without conductive metal coatings. Qualitative elemental analysis was performed by energy dispersive X-ray spectroscopy (EDS), which allows the simultaneous semi-quantification of different elements in the sampled seeds. In total, several dozens of measurements of different internal and external seed areas were tested (~ 4 per seed).

Surface roughness was measured using a Sensofar S-Neox non-contact 3D optical profiler. Three seeds per studied population were measured (12 in total). One-fourth of the surface of each seed was analyzed. A three-dimensional view was generated. This view shows a complex surface structure, which allowed us to assess the morphology of the surface from different angles. In the analysis, the overall form was removed from the surface to calculate the local surface roughness of the seeds. The “Sa” parameter (arithmetical mean height) was taken into account. According to ISO 25178 standards, in which surface texture parameters are described, the Sa parameter is an extension of the “Ra” parameter (arithmetical mean height of a line) to a surface. The Sa parameter is generally used to evaluate surface roughness.

The data on the parameters describing seed size, shape and structure were included in the electronic supplemental material (Tables S1, S2).

### Statistical analysis

The data were analyzed with SPSS software (ver. 24.0; IBM Corp. 2016). The data were analyzed with the use of generalized linear mixed models (GLMMs) function, although the random factor was not included in the models. It should be noted that the GLMM function without specifying a random factor in SPSS is equivalent to the General Linear Model (GLM) function and produces exactly the same results. We used the GLMM function since pairwise contrasts (needed in for comparisons between the studied species from different populations) are not available at the GLM function in SPSS.

Linear models were used for the interval target variables (the frequency of the viable seeds from the TZ test, the Ss, the circularity, the aspect ratio and the Sa parameter from the roughness test), and negative binomial regression was used for numerical data (the number of sunken seeds in both the still and turbid water experiments). We applied the Satterthwaite approximation in the TZ and surface roughness tests.

The same type of statistical model was used for the still and turbid water experiments, corresponding to the three phases: initial (hereafter “Day 0” or “Second 20”), intermediate (hereafter “Day 11/12” or “Second 960”) and final (hereafter “Day 62/64” or “Second 12,480”). In the still water experiment, the 11th and 62nd days were for the Insubrian populations of both species, and the 12th and 64th days for the Cracow and Istria populations. In each of the cases, the number of sunken seeds was the target variable, and the fixed effects were species and population age (two categories: younger, older). The interaction between species and population age was also included in each of the tests.

In the TZ test, only seeds previously used in the still water experiments were included because we assumed that the period of time over which the turbid water tests were conducted was too short to kill the seeds. The seeds were divided into 16 categories according to the following criteria: species (*I. balfourii*, *I. glandulifera*), population age (younger populations, older populations), floating ability (1—floating seeds, 2—sunken seeds), and mildew occurrence (mildewed, non-mildewed). For each of the categories, the frequency of viable seeds was calculated, arcsine transformed and used as the target variable in the statistical model. The fixed effects were species, population age, floating ability and their interactions (species × population age, species × floating ability, population age × floating ability, species × population age × floating ability). A parallel test with the occurrence of mildew instead of the floating ability was also carried out.

The statistical model for the Ss assumed a gamma distribution of the target variable. The model included two fixed effects (species, population age) and one interaction (species × population age). The circularity, aspect ratio and surface roughness were tested using a linear model in which species, population age and the interaction between these two variables were included (as in the model for the Ss).

## Results

### The ability of the seeds to float in still water

**Fig. 3:**
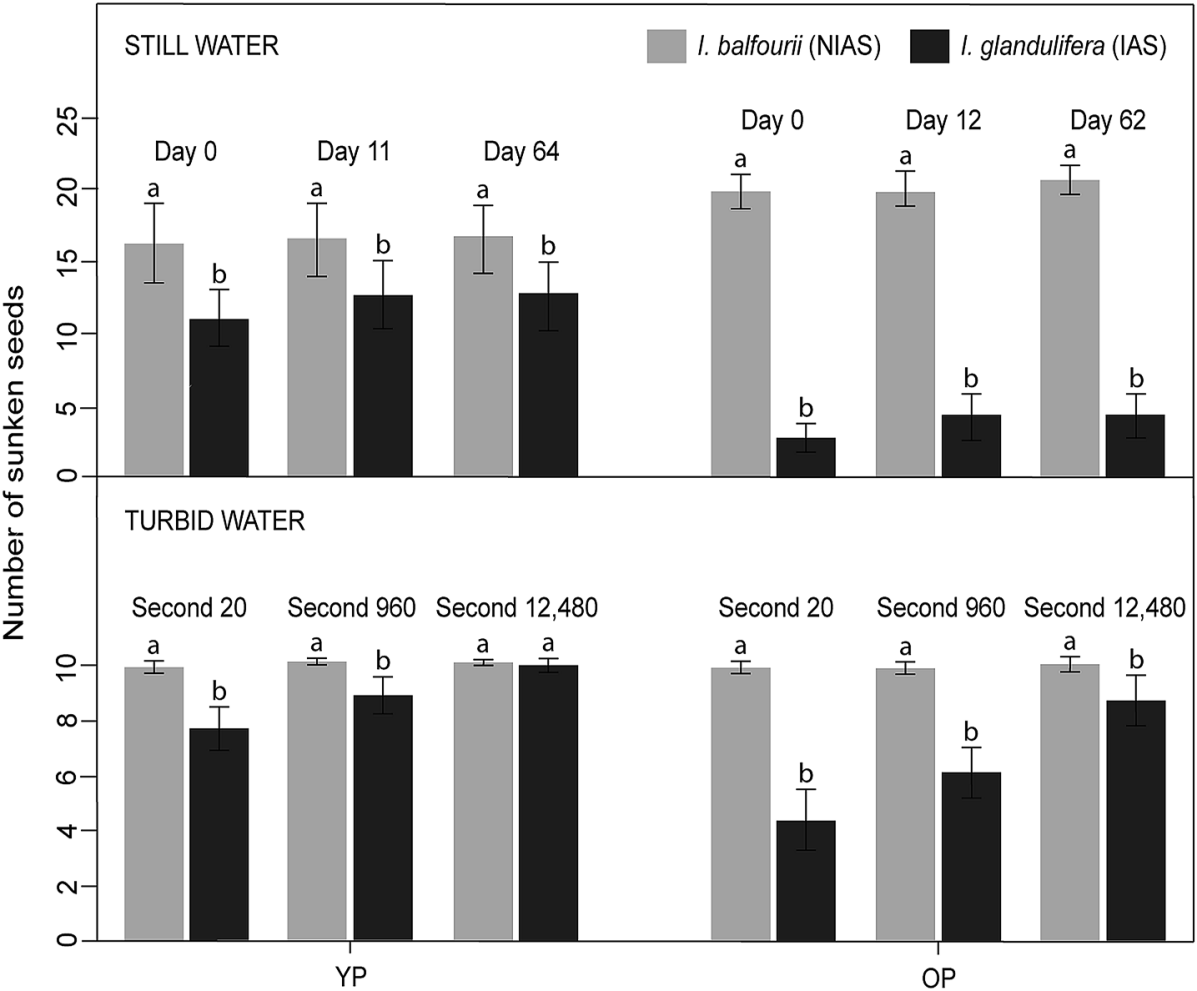
The number of sunken seeds of the non-invasive alien species (‘NIAS’) *Impatiens balfourii* and the invasive alien species (‘IAS’) *I. glandulifera* in the still and turbid water experiments (± SE). The seeds were collected from populations differing in age: ‘YP’ means the younger population, and ‘OP’ means the older population. The three phases of the still (“Day 0”, “Day 11/12”, and “Day 62/64”) and turbid water experiments (“Second 20”, “Second 960”, and “Second 12,480”; see “Materials and methods”) are shown. The letters above the T-bars indicate significant differences between the species within particular experiments

Comparisons of the species revealed that the seeds of *I. balfourii* sank more frequently than the seeds of *I. glandulifera* (Tables 2, 3). However, the results were driven by mainly the interactions between the species and population age, which was substantial in the initial, intermediate and final phases (Table 3, interaction: *p* < 0.001 in all phases). The differences between the species were greater when the seeds were derived from older populations (contrasts: *p* < 0.001 in all phases; Fig. 3) than when they were derived from younger populations (contrast for Day 0: *p* < 0.001; contrast for Day 11/12: *p* = 0.018; contrast for Day 62/64: *p* = 0.012; Fig. 3).

**Table 2:** The number of sunken seeds of the non-invasive alien species (‘NIAS’) *I. balfourii* and the invasive alien species (‘IAS’) *I. glandulifera* in the still and turbid water experiments

Phase	*I. balfourii* (NIAS)		*I. glandulifera* (IAS)	
	Istria (YP)	Insubria (OP)	Cracow (YP)	Insubria (OP)
Day 0	163	199	111	28
Day 11/12	164	201	127	42
Day 62/64	165	215	126	43
Second 20	99	99	77	44
Second 960	100	99	88	61
Second 12,480	100	99	99	86

The seeds were collected from populations differing in age: ‘YP’ means younger population, and ‘OP’ means older population. The three phases of the still (“Day 0”, “Day 11/12”, and “Day 62/64”) and turbid water experiments (“Second 20”, “Second 960”, “Second 12,480”; see “Materials and methods”) are shown

**Table 3:** The results of the GLMM for the number of sunken seeds in the still water experiment

Effect	*F*	df	*p*
Day 0			
Species	156.34	37	< 0.001
Population age	39.43	37	< 0.001
Species × Population age	70.67	37	< 0.001
Day 11/12			
Species	101.16	37	< 0.001
Population age	24.87	37	< 0.001
Species × Population age	52.33	37	< 0.001
Day 62/64			
Species	105.51	35	< 0.001
Population age	22.68	35	< 0.001
Species × Population age	52.67	35	< 0.001

The model compares the seed floating ability of the non-invasive alien species (‘NIAS’) *I. balfourii* and the invasive alien species (‘IAS’) *I. glandulifera* from populations differing in age. Three phases of the experiment are shown: “Day 0”, “Day 11/12” and “Day 62/64” (see “Materials and methods”)

When each species was considered separately, populations that differed in age also had different floating abilities (contrasts for *I. glandulifera*: *p* < 0.001 in all phases; contrasts for *I. balfourii*: *p* < 0.02 in all phases; Fig. 3). Moreover, the trend in the floating ability was opposite in the two species—seeds from the younger population of *I. balfourii* floated better than seeds from its older population, whereas seeds of *I. glandulifera* from the older population floated better than seeds from its younger population. These patterns were consistent during each phase of the experiment (Table 3, Fig. 3).

### The ability of seeds to float in turbid water

The results showed significant differences between the two species (Tables 2, 4), and as in the case of the still water tests, the results were driven by interactions between the species and population age in all phases. The seeds of *I. balfourii* sank more frequently than the seeds of *I. glandulifera* at the beginning of the experiment and in the intermediate phase (contrast for Second 20: *p* < 0.001; contrast for Second 960: *p* = 0.008; Fig. 3), while in the final phase (Second 12,480, Fig. 3), there were no differences between the seeds of the two species from the younger populations (contrast: *p* = 0.8; Fig. 3). The results obtained for the older populations were consistent throughout the experiment, with a higher number of sunken seeds for *I. balfourii* (contrasts: *p* < 0.001 in all phases; Fig. 3).

**Table 4:** The results of the GLMM for the number of sunken seeds in the turbid water experiment

Effect	*F*	df	*p*
Second 20			
Species	115.22	37	< 0.001
Population age	31.98	37	< 0.001
Species × Population age	31.98	37	< 0.001
Second 960			
Species	80.65	37	< 0.001
Population age	30.52	37	< 0.001
Species × Population age	27.35	37	< 0.001
Second 12,480			
Species	10.25	37	0.003
Population age	10.25	37	0.003
Species × Population age	7.70	37	0.009

The model compares the seed floating ability of the non-invasive alien species (‘NIAS’) *I. balfourii* and the invasive alien species (‘IAS’) *I. glandulifera* from populations differing in age. The three phases of the experiment are shown: “Second 20”, “Second 960” and “Second 12,480” (see “Materials and methods”)

When each species was considered separately, the results showed significant differences between the younger and older *I. glandulifera* populations (contrasts: *p* < 0.001 in all phases; Fig. 3). However, there were no significant differences in the floating ability of *I. balfourii* seeds between the two populations differing in age (contrasts: *p* > 0.7 in all phases; Fig. 3).

### Tetrazolium (TZ) test

**Fig. 4:**
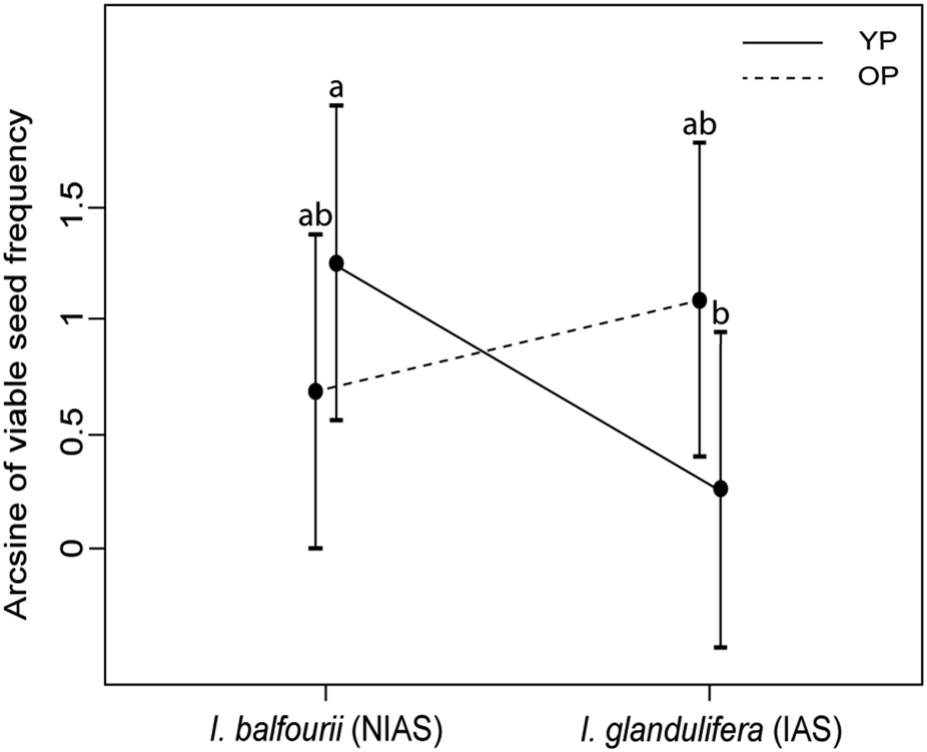
Frequency of viable sunken seeds of the non-invasive alien species (‘NIAS’) *Impatiens balfourii* and the invasive alien species (‘IAS’) *I. glandulifera* in the tetrazolium (TZ) test. Estimated mean arcsine-transformed frequencies (± confidence intervals) are shown for both species. The seeds were collected from populations differing in age: ‘YP’ means the younger population, and ‘OP’ means the older population. The letters above the T-bars indicate significant differences between the species

In the seed viability model, only the interaction among floating ability, population age and plant species was significant (Table 5; Fig. 4). The significance of the overall result was determined by divergence among the sunken seeds with no floating ability (Fig. 3). The results differed for the the two species in that the number of viable seeds in the younger populations was significantly higher for *I. balfourii* (contrast: SE = 0.42, *t* = 2.35, df = 8, *p* = 0.047; Fig. 4), while the results in the older populations seemed to be higher only for *I. glandulifera* (contrast: *p* > 0.1, Fig. 4). At the same time, the frequency of viable seeds with the ability to float did not differ between the *Impatiens* species (the result was not statistically significant at *p* > 0.1).

**Table 5:** Results of the tetrazolium (TZ) test (with the frequency of viable seeds as a target variable)

Effect	*F*	df	*p*
Species	1.69	9	0.230
Population age	0.22	9	0.651
Species × Population age	0.15	9	0.704
Floating ability	0.00	9	0.987
Species × Floating ability	0.01	9	0.923
Population age × Floating ability	0.03	9	0.874
Species × Population age × Floating ability	8.43	9	0.020

The model compared the non-invasive alien species (‘NIAS’) *I. balfourii* and the invasive alien species (‘IAS’) *I. glandulifera* from populations differing in age and included their seed floating ability. The data for the seeds previously used in the still water experiment were used in the model

A similar test of mildew occurrence instead of floating ability was also carried out. Mildew was recorded on 29.6% of the seeds of both species. However, none of the obtained results were significant (*p* > 0.1 for all variables and interactions).

### Seed surface index (Ss) and shape descriptors

**Fig. 5:**
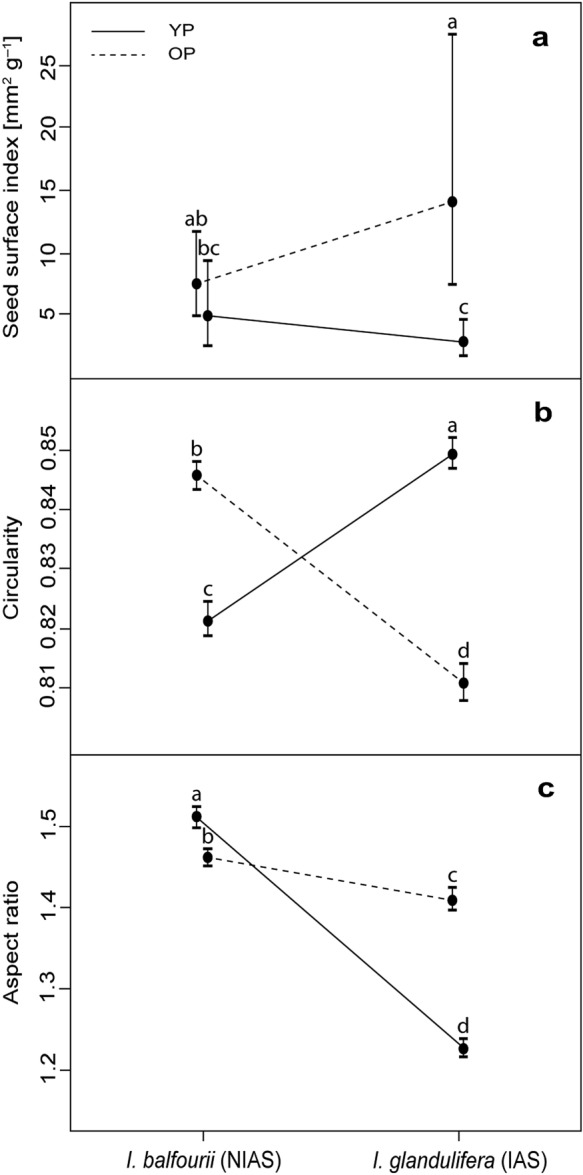
Estimated mean values (± confidence intervals) of the seed surface index (Ss; plot **a**), circularity (plot **b**) and aspect ratio (plot **c**) of seeds are shown for both species. Varying levels of invasiveness of the two species are indicated: ‘NIAS’—the non-invasive alien species, ‘IAS’—the invasive alien species. The seeds were collected from populations differing in age: ‘YP’ means the younger population, and ‘OP’ means the older population. The letters above the T-bars indicate significant differences between the species

Although the “species” variable was non-significant in the model (Table 6), the tests of the seeds of each species originating from different populations (see the interaction between species and population age, Table 6; Fig. 5a) revealed that the Ss of *I. glandulifera* seeds was higher in the older population than in the younger population (contrast: SE = 4.5, *t* = 2.48, df = 20, *p* = 0.02; Fig. 5a). In the case of *I. balfourii*, there were no differences between the older and younger populations (contrast: *p* = 0.2; Fig. 5a).

**Table 6:** Results of the models of the seed surface index (Ss), circularity and aspect ratio (see “Materials and methods”)

Effect	*F*	df	*p*
Ss index			
Species	0.03	21	0.86
Population age	13.58	21	0.001
Species × Population age	4.52	21	0.046
Circularity			
Species	13.22	6212	< 0.001
Population age	54.98	6212	< 0.001
Species × Population age	1022.30	6212	< 0.001
Aspect ratio			
Species	886.02	6212	< 0.001
Population age	139.67	6212	< 0.001
Species × Population age	423.25	6212	< 0.001

The models compared the non-invasive alien species (‘NIAS’) *I. balfourii* and the invasive alien species (‘IAS’) *I. glandulifera* from populations differing in age

In the models of the circularity and aspect ratio, all variables and interactions were statistically significant (Table 6). In both models, the interactions showed that the circularity and the ratio between seed axes differed between the two species and within the same species between the populations of different ages. The analysis showed that the circularity was lower for the populations of both species with the greater floating potential (contrast for populations of *I. balfourii*: SE = 0.001, *t* = − 18.02, df = 6211, *p* < 0.001; for *I. glandulifera*: SE = 0.001, *t* = 26.91, df = 6211, *p* < 0.001; Fig. 5b). Moreover, the aspect ratio increased with the floating potential (contrast for populations of *I. balfourii*: SE = 0.008, *t* = 6.42, df = 6211, *p* < 0.001; for *I. glandulifera*: SE = 0.008, *t* = − 22.13, df = 6211, *p* < 0.001; Fig. 5c). These results showed that the oblong seeds of both *Impatiens* species floated better than the rounded ones.

### Seed structure assessment

**Fig. 6:**
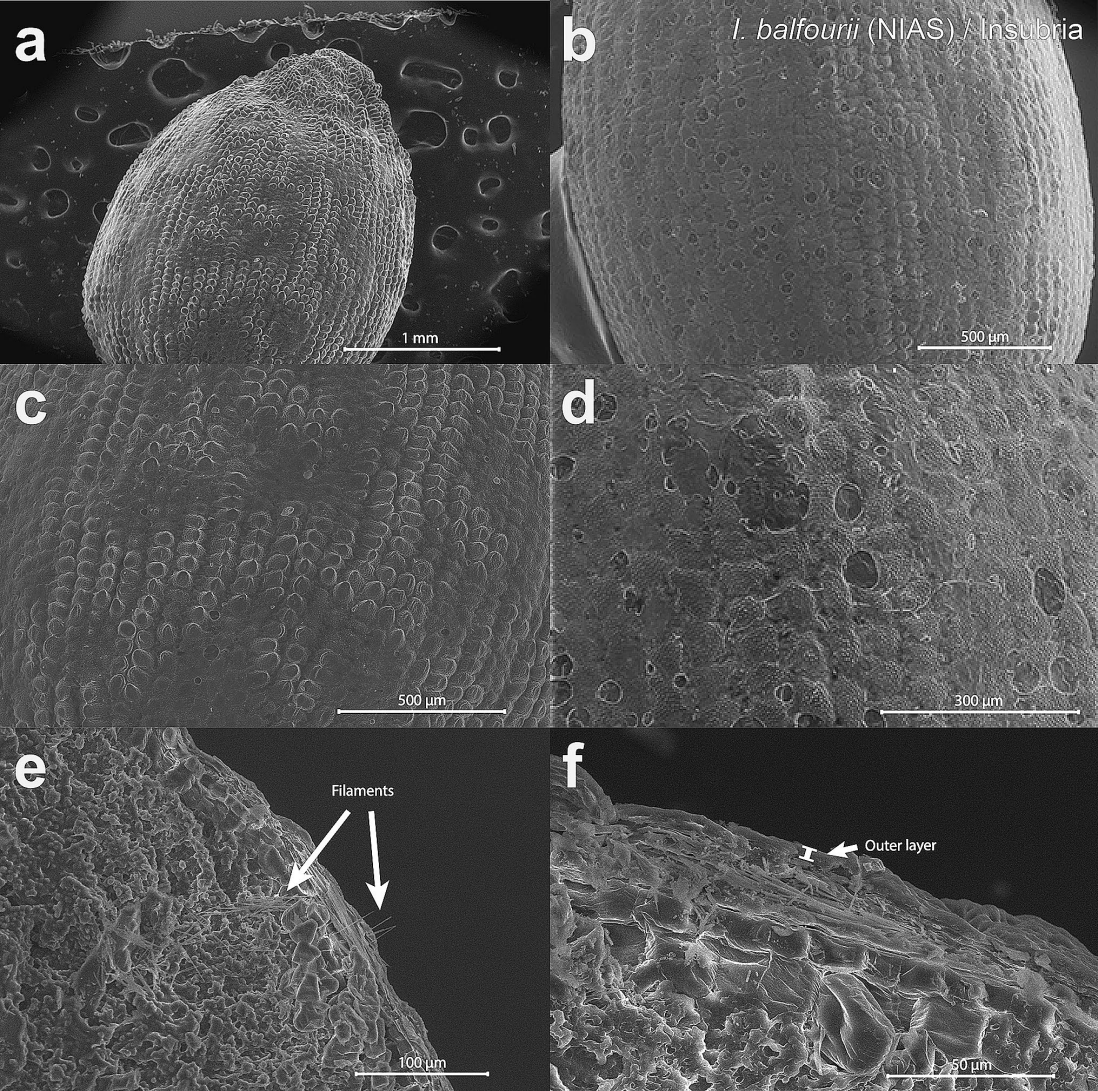
SEM micrographs of the examined seeds of the non-invasive alien species (‘NIAS’) *Impatiens balfourii* from the older population in Insubria. The images show surfaces (**a**–**d**) and seed coat cross-sections (**e**, **f**). The filaments are indicated by arrows in the image in **e**. The outer layer of the seed coat is presented by the image in **f**

**Fig. 7:**
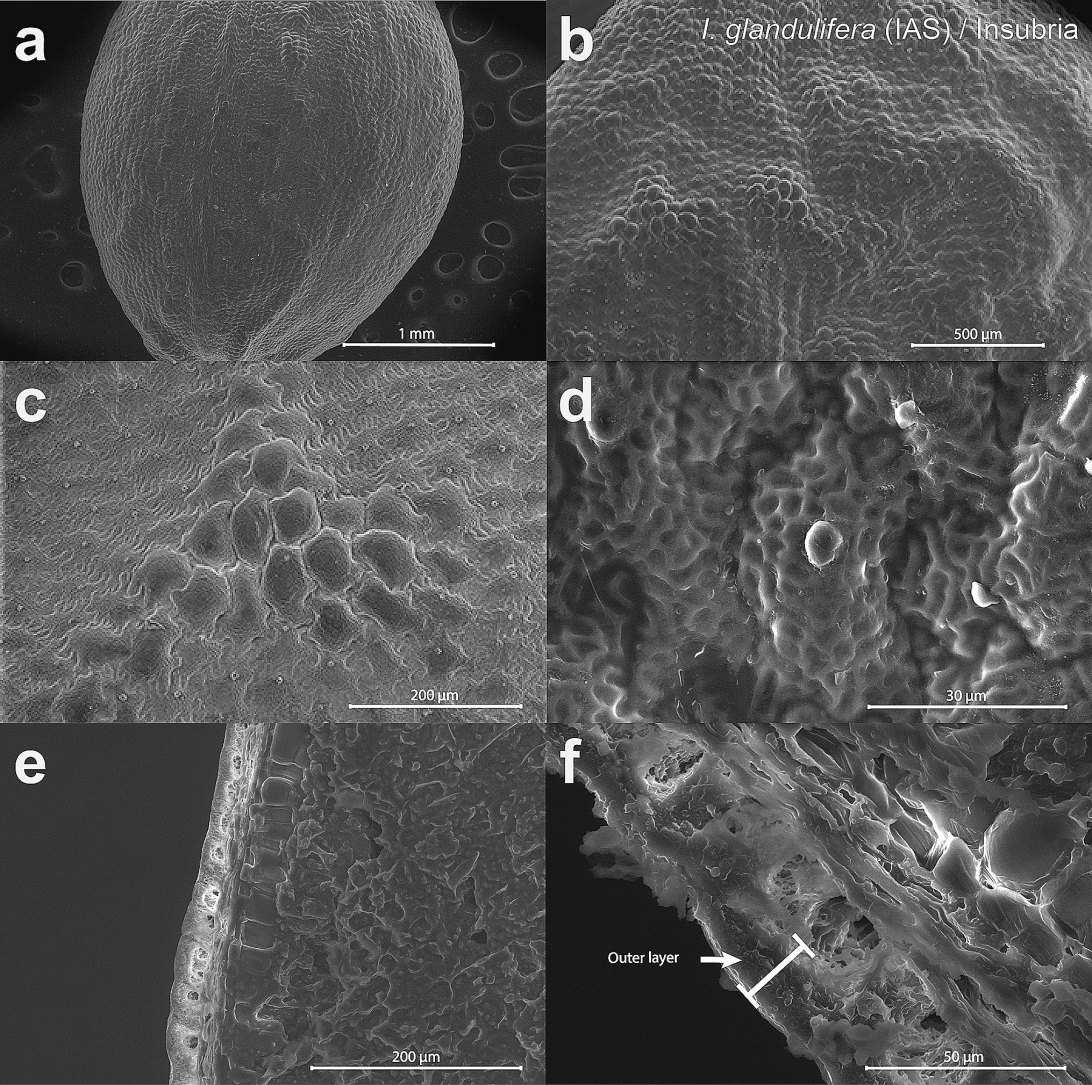
SEM micrographs of the examined seeds of the invasive alien species (‘IAS’) *Impatiens glandulifera* from the older population in Insubria. The images show surfaces (**a**–**d**) and seed coat cross-sections (**e**, **f**). The outer layer of the seed coat is presented by the image in **f**

**Fig. 8:**
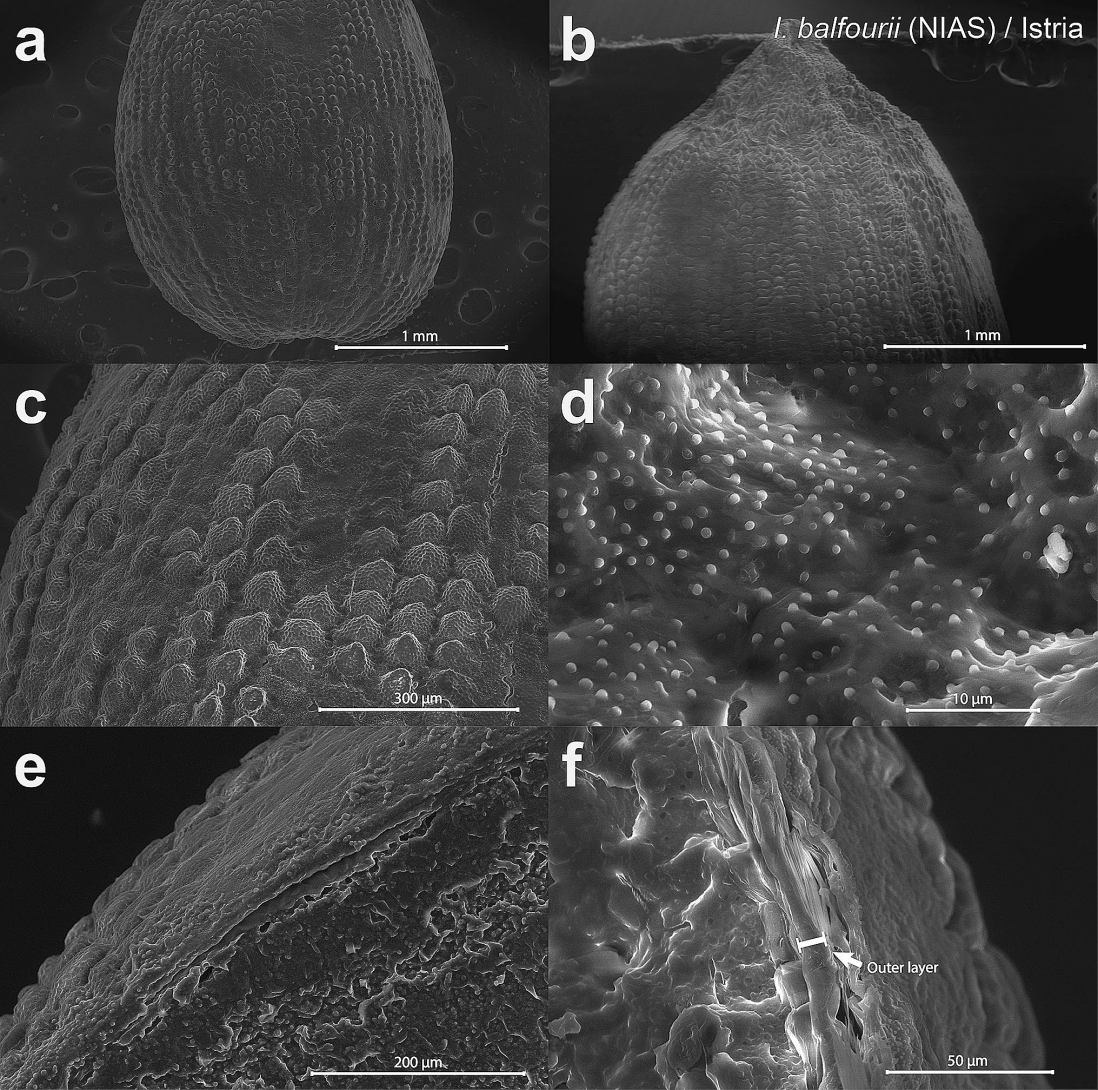
SEM micrographs of the examined seeds of the non-invasive alien species (‘NIAS’) *Impatiens balfourii* from the younger population in Istria. The images show surfaces (**a**–**d**) and seed coat cross-sections (**e**, **f**). The outer layer of the seed coat is presented by the image in **f**

**Fig. 9:**
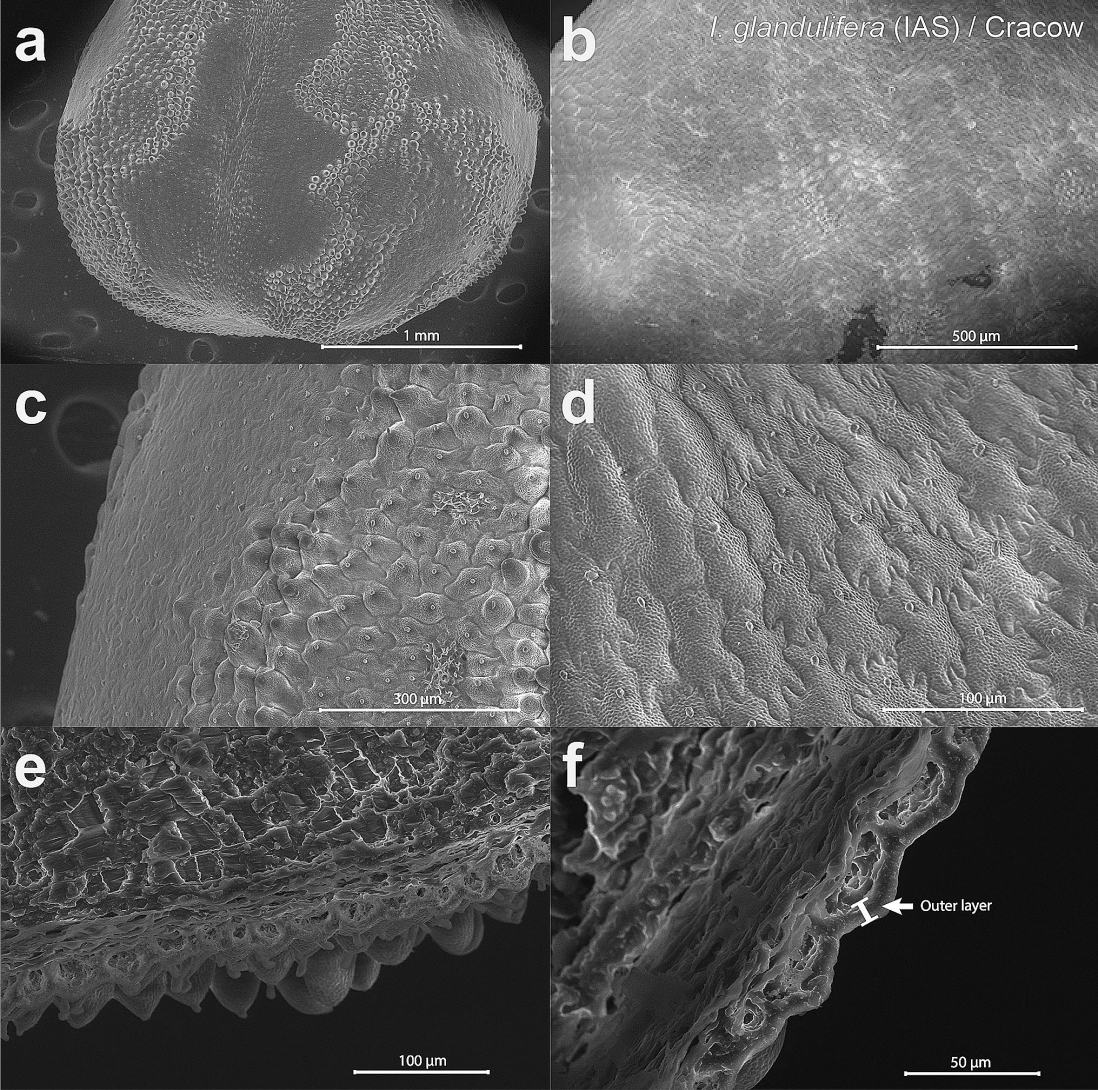
SEM micrographs of the examined seeds of the invasive alien species (‘IAS’) *Impatiens glandulifera* from the younger population in Cracow. The images show surfaces (**a**–**d**) and seed coat cross-sections (**e**, **f**). The outer layer of the seed coat is presented by the image in **f**

No straightforward differences that could potentially affect the floating ability were found in the seed surface structures of the two *Impatiens* from any of the studied localities. SEM (Figs. 6, 7, 8, 9a–d) assessments revealed that the seed surface could be either smooth or irregular, even within the same population. Similarly, analysis of the Sa parameter, representing the surface roughness, yielded non-significant results (*p* > 0.1 for all variables and the interaction). The only exception was the large “bald” areas of smooth surfaces in the Cracow population of *I. glandulifera* (Fig. 9a, b), which significantly decreased the ability of the seeds to float. Such bald areas were not detected in the SEM analysis of *I. glandulifera* seeds from Insubria (Fig. 7a, b).

Substantial differences, however, were found in the seed cross-section assessments (Figs. 6, 7, 8, 9e, f). Regardless of the population age, *I. glandulifera* had more air-filled cavities inside its seed coats than *I. balfourii*. Differences in the seed cross-sections related to population age were also substantial in the outer layer of the coat that wraps around the air-filled cavity system in both species. In the case of *I. glandulifera* from Insubria, the outer layer was approximately four times thicker (Fig. 7f) than that of seeds from Cracow (Fig. 9f). The width of this layer in *I. balfourii* seeds from Istria (Fig. 8f) was similar to that of *I. glandulifera* seeds from Cracow and twice as thick as that of *I. balfourii* seeds from Insubria (Fig. 6f). Thus, the width of the outer layer in seeds of both *Impatiens* species was higher in seeds with better floating ability.

The analysis of the element content showed that the outer layer contains a greater abundance of calcium and oxygen containing compounds than deeper parts of the coat. In the outer layer of the seeds of both species from all populations, filaments were also present (Fig. 6e) and contained the highest amounts of calcium and oxygen containing compounds among all tested parts of the seeds.

## Discussion

The presented results indicate that the differences in invasiveness between *I. glandulifera* and *I. balfourii* may be driven by differences in the floating ability of their seeds. The overall result of the still and turbid water tests was that seeds of the non-invasive *I. balfourii* floated less well than those of the invasive *I. glandulifera.* However, it should be noted that in the older populations of both species, the seeds of *I. balfourii* floated less well than the seeds of *I. glandulifera*, whereas in the younger populations, the differences between the seeds of the two species were considerably reduced. These results indicate that the floating ability of *I. balfourii* seeds may increase over time after its introduction into a given area, while in the case of *I. glandulifera,* this ability may gradually decrease. Taking into account that *I. balfourii* was introduced 60 years later than its invasive counterpart (Adamowski 2009), our results fit the concept that some alien species pass through a lag phase before they become invasive, which is well established in biological invasion studies. The factors that lead to the cessation of such dormancy include changes in climate and habitat, the opening of new transport corridors, the overcoming of Allee effects at low densities, and interspecific interactions of newly introduced alien species (Crooks 2011). Nevertheless, it should also be stressed that the seeds used in this study were influenced by differences in maternal environmental conditions, as the seeds originated from field localities (not from common garden conditions) and the younger populations of the two species (Istria and Cracow) were distant from each other. The two species co-occurred under comparable conditions only in Insubria. Thus, the obtained differences between the two *Impatiens* populations of different ages should be interpreted with caution.

The tests of seed viability allowed us to estimate seed survival after a long period of submergence in water. We found that, for both species, more viable seeds were recorded from the populations in which seeds generally floated better. This result was obtained for sunken seeds but not floating seeds. This indicates that while the chances of survival on the water surface were equal among all studied populations, sunken seeds were more resistant to long submergence if they came from populations in which the seeds floated better. It should also be stressed that mildew occurrence, also included in the TZ assessment, did not affect any of the tested seed characteristics.

Insight into the mechanisms determining the differences detected in the floating ability tests was provided by the analysis of the Ss, shape descriptors and seed structure. We assumed that floating ability would increase with the Ss, but the obtained results were consistent with this hypothesis for only *I. glandulifera*. For this species, the index value was significantly lower for the younger population (Cracow and its surrounding areas), which was characterized by seeds with a lower floating ability. This relatively young population is widespread and expanding and thus seems to be in good condition (Zając et al. 2011). An explanation may be that numerous populations of this species in areas near the invasion front, e.g., in Cracow, 100 km north of the invasion boundary (Zając et al. 2011), may reduce their investment in floating ability and reallocate the saved resources to other characteristics to maximize fitness (e.g., fertility). At the same time, individuals from the two populations of *I. balfourii* did not differ; thus, they did not invest in seed size. Therefore, in contrast to results of the previous studies on *Impatiens* (Chmura et al. 2013; Čuda et al. 2016), our findings indicate that smaller seeds are not always associated with less broadly distributed species. On the other hand, the results of the analyzes of seed shape and structure indicate that in the two *Impatiens* species, these factors determine their floating ability. We found that the oblong seeds floated better than the rounded seeds, which was consistent for both *Impatiens* species. In the structure analysis, we revealed large “bald” areas of smooth surfaces on seeds of *I. glandulifera* from the population with a lower floating potential. Thus, it is highly probable that the reduction in surface roughness decreased the floating potential of the studied seeds. The factors with the strongest potential to determine the floating ability of both species were found in the cross-section assessment. First, *I. glandulifera* had considerably more cavities inside its seed coat than *I. balfourii*. It is clear that this air-filled “cavity system” determines the ability of the seeds to float in water. Moreover, regardless of the studied species, we found that the uniform outer layer that wraps around the cavity system was thicker in populations with a better floating ability and thinner in populations with a lower floating potential.

Further examination of the outer layer by EDS revealed that it contained significantly more calcium and oxygen-containing compounds than the internal parts of the seed coat. Calcium is essential to the stability of the structure, while oxygen plays a crucial role in seed germination and seedling establishment (Liu et al. 2012; Naylor and Prentice 1986). At the same time, water is a barrier to oxygen diffusion, and seed vigor is reduced by excess water in the soil, for example, during long periods of rainfall or flooding (Liu et al. 2012; Naylor and Prentice 1986). Thus, it can be assumed that the outer layer of seeds of the studied *Impatiens* species, in which the levels of calcium and oxygen were high, may also protect the seeds during floating, as the seeds may persist in good conditions even after long periods of submergence in water.

Because seeds become impermeable during the later stages of maturation and this process differs among plant species (Souza and Marcos-Filho 2001), in the present study, only fully developed, mature seeds were included. We suppose that the level of water penetration into the seed coat decreases with increasing quality of the outer layer, and in fact, this may be the most important factor that determines the floating ability of seeds. Therefore, better-equipped seeds (e.g., *I. glandulifera* from Insubria with the thickest layer) are capable of floating longer on the water surface, which was confirmed in the presented tests. Consequently, such seeds may be dispersed over longer distances than those with a thin outer layer. Another advantage of a high-quality outer layer may be more efficient competition during prolonged periods of excess water levels (e.g., floods) or in floodplain areas, which are known to be among the most heavily invaded habitats (Truscott et al. 2006). The success of invasive alien species could, therefore, be explained by their higher germination success facilitated by a thick outer layer, effectively protecting them from excessive water penetration.

An additional advantage of the present study is the fact that *I. balfourii* is a poorly studied species (ISI Web Of Science 2019), which may be an effect of its relatively low invasiveness (in terms of a small range conquered after introduction and no documented negative impacts). However, although such non-invasive alien species may not pose a serious imminent threat to native biodiversity or local economies, studying them is particularly important because they may yield insights into the mechanisms underlying the development of invasiveness, which are important for the theoretical aspects of biological invasion as well as the development of practical measures to mitigate their impacts (Ugoletti et al. 2011; van Kleunen et al. 2010).

## Conclusions

In this study, we showed that the ability of seeds to float may promote the invasiveness of alien plant species. A comparative examination of the floating ability of seeds of non-invasive *I. balfourii* and invasive *I. glandulifera* provides new insights into the mechanisms of alien annual plant spread. The results can be used to predict future invasion corridors of both *Impatiens* species and design strategies for their eradication and management.

Our results may also suggest that *I. balfourii* has undergone changes in terms of the floating ability of its seeds. It can therefore be assumed that although this species is commonly regarded as a poor disperser (Najberek et al. 2017), the lag phase that it has been in may end in the near future. Consequently, the rate of its spread may significantly increase, and it may become a truly invasive alien species in Europe. This result should be taken into account in analyzes of the invasive potential of alien species that have not yet entered the phase of rapid spread. Identification of species that may become invasive is a key element for designing strategic plans to combat them, which necessitates the identification of invasive characteristics that are targets of post-introduction changes.

In contrast, we showed that the ability of *I. glandulifera* seeds to spread via rivers is greater in the older population. This knowledge can help efficiently predict invasion scenarios for other harmful or potentially harmful alien plant species that spread along water courses because it indicates that widely distributed alien plant invaders may decrease the rate of their spread as the invasion becomes well advanced (Lee 2011; Schupp 2011).

According to the latest data on the non-invasive *I. balfourii* (Najberek et al. 2020), the future spread of this species may be strongly associated with streams. As *I. glandulifera* is already widely distributed along rivers throughout Europe, further comparisons of seed floating potential between these two closely related *Impatiens* species are needed. Using seeds from common garden cultivation would be particularly interesting, as it would indicate whether the differences in floating ability between populations of differing ages are driven by genetic shifts or by maternal environmental conditions.

## Electronic supplementary material

Below is the link to the electronic supplementary material.

Supplementary file1 (PDF 129 kb)
