# Supplementary material for: The ability of seeds to float with water currents contributes to the invasion success of Impatiens balfourii and I. glandulifera
Source: J Plant Res. 2020 Jul 3;133(5):649–64. doi: 10.1007/s10265-020-01212-0 (PMC7429548; doi:10.1007/s10265-020-01212-0)
Supplement: Supplementary file 1 — Supplementary file1 (PDF 129 kb) [file 10265_2020_1212_MOESM1_ESM.pdf]

Electronic Supplementary Material

Title: The ability of seeds to float with water currents contributes to the invasion success of *Impatiens balfourii* and *I. glandulifera*

Najberek Kamil[1]*, Olejniczak Paweł[1], Berent Katarzyna[2], Gąsienica-Staszeczek Magdalena[3], Solarz Wojciech[1]

* Corresponding author. Email: najberek@iop.krakow.pl

Affiliations of authors:

[1] Institute of Nature Conservation, Polish Academy of Sciences, al. Adama Mickiewicza 33, 31-120 Kraków, Poland

[2] AGH University of Science and Technology, Academic Centre for Materials and Nanotechnology, al. Adama Mickiewicza 30, 30-059 Kraków, Poland

[3] Wojdyły 13/9, 34-500 Zakopane

Table S1. Data on the physical characteristics of the seeds from the non-invasive alien species ('NIAS') *Impatiens balfourii* and the invasive alien species ('IAS') *I. glandulifera* populations. The data were used to assessed the seed size, circularity and aspect ratio. The table includes average values (± SD) of the seed area, seed weight, seed perimeter and the length of major and minor seed axes.

Population age	Area [mm^2]	Perimeter [mm]	Major axis [mm]	Minor axis [mm]	Circularity	Weight [g]	Ss Index [mm^2/g]
***I. balfourii* (NIAS):**							
Younger population	4.42 ± 0.81	8.18 ± 0.73	2.89 ± 0.24	1.93 ± 0.24	0.82 ± 0.03	1.60 ± 0.51	3.46 ± 2.33
Older population	4.05 ± 0.71	7.73 ± 0.64	2.72 ± 0.24	1.89 ± 0.24	0.85 ± 0.04	1.54 ± 0.90	4.16 ± 3.87
***I. glandulifera* (IAS):**							
Younger population	9.23 ± 2.09	11.61 ± 1.33	3.75 ± 7.25	3.09 ± 0.44	0.85 ± 0.03	3.95 ± 1.11	2.56 ± 1.04
Older population	9.13 ± 2.15	11.82 ± 1.39	3.99 ± 0.44	2.88 ± 0.44	0,81 ± 0.05	1.16 ± 0.35	9.99 ± 8.18

Table S2. Data on the roughness of the seeds from the non-invasive alien species ('NIAS') *Impatiens balfourii* and the invasive alien species ('IAS') *I. glandulifera* populations. Sa – Arithmetical mean height, Sq – Root mean square height, Sp – Maximum peak height.

Population age	Seed no.	Sa [µm]	Sq [µm]	Sp [µm]
I. balfourii **(NIAS):**				
	1	6.78	8.77	318.15
Younger population	2	8.25	10.04	30.98
	3	8.83	11.55	39.81
	1	11.93	14.89	307.84
Older population	2	4.20	5.20	18.68
	3	7.66	9.45	39.21
I. glandulifera **(IAS):**				
	1	6.89	9.09	32.28
Younger population	2	12.73	15.97	39.37
	3	6.94	8.67	35.64
	1	18.94	23.66	565.86
Older population	2	10.56	13.10	34.36
	3	5.85	7.36	31.05
